# Protective effect of recombinant staphylococcal enterotoxin A entrapped in polylactic-co-glycolic acid microspheres against *Staphylococcus aureus *infection

**DOI:** 10.1186/1297-9716-43-20

**Published:** 2012-03-19

**Authors:** Liben Chen, Shuang Li, Zhengfang Wang, Ruilong Chang, Jingliang Su, Bo Han

**Affiliations:** 1 Department of Clinical Medicine, College of Veterinary Medicine, China Agricultural University, Beijing 100193, China; 2 Beijing Veterinary Diagnostic Institute, Beijing 100101, China; 3 Key Laboratory of Animal Epidemiology and Zoonosis of the Ministry of Agriculture, College of Veterinary Medicine, China Agricultural University, Beijing 100193, China; 4 Department of Chemistry and Biochemistry, Ohio University, Athens 45701, OH, USA

## Abstract

*Staphylococcus aureus *is an important cause of nosocomial and community-acquired infections in humans and animals, as well as the cause of mastitis in dairy cattle. Vaccines aimed at preventing *S. aureus *infection in bovine mastitis have been studied for many years, but have so far been unsuccessful due to the complexity of the bacteria, and the lack of suitable vaccine delivery vehicles. The current study developed an *Escherichia coli *protein expression system that produced a recombinant staphylococcal enterotoxin A (rSEA) encapsulated into biodegradable microparticles generated by polylactic-co-glycolic acid (PLGA) dissolved in methylene chloride and stabilized with polyvinyl acetate. Antigen loading and surface properties of the microparticles were investigated to optimize particle preparation protocols. The prepared PLGA-rSEA microspheres had a diameter of approximately 5 μm with a smooth and regular surface. The immunogenicity of the PLGA-rSEA vaccine was assessed using mice as an animal model and showed that the vaccine induced a strong humoral immune response and increased the percent survival of challenged mice and bacterial clearance. Histological analysis showed moderate impairment caused by the pathogen upon challenge afforded by immunization with PLGA-rSEA microspheres. Antibody titer in the sera of mice immunized with PLGA-rSEA microparticles was higher than in vaccinated mice with rSEA. In conclusion, the PLGA-rSEA microparticle vaccine developed here could potentially be used as a vaccine against enterotoxigenic *S. aureus*.

## Introduction

*Staphylococcus aureus (S. aureus) *is an important cause of nosocomial and community-acquired infections in humans and animals, and economic loss in animal husbandry, such as mastitis in dairy cattle [1]. *S. aureus *can provoke clinical mastitis but more frequently causes subclinical infections that tend to become chronic and difficult to eradicate by conventional antimicrobial therapies [2,3].

The frequent incapacity of both the antibiotics and immune response to prevent infection and destroy the pathogen in the intramammary environment explains why *S. aureus *bovine mastitis constitutes a major challenge to dairy producers [4]. Multidrug-resistant *S. aureus *infections continue to increase, and some strains respond to few, if any, conventional antibiotic therapies. Hence, interest in immunotherapeutic strategies, either passive or active, has seen resurgence in recent years. The pathogenicity of *S. aureus *results from structures that allow it to avoid phagocytosis, the production of enzymes and toxins, including exotoxins such as staphylococcal enterotoxins (SE), toxic shock syndrome toxins (TSST), exoenzymes, adhesins, and numerous cell-associated components that either directly cause disease or facilitate tissue penetration and immune cell recruitment [5,6]. Therefore, application of efficacious vaccine is one of the most important prophylactic measures against bovine mastitis.

Several studies have recently focused on *S. aureus *toxins as vaccine targets [7-9]. SE, which are bacterial superantigens (sAg) produced by *S. aureus*, play an important role in establishing and maintaining infection [5]. Immunization with recombinant or mutant staphylococcal enterotoxin A (SEA) [10], staphylococcal enterotoxin B (SEB) [11-13], and TSST-1 [12,14] can elicit neutralizing antibodies against wild-type SE, and has been shown to protect mice, rabbits, and monkeys against lethal shock induced by wild-type superantigenic toxins. Hu et al. have investigated a double-mutant staphylococcal enterotoxin C (SEC) [15], that was devoid of superantigenic activity, as an intranasal vaccine for protection against *S. aureus *challenge in mice. Other groups recently studied a recombinant staphylococcal enterotoxin C mutant in lactating dairy cattle and found that vaccinated cattle had lower milk somatic cell counts and lower numbers of intra-mammary infection than unvaccinated controls [16,17].

Modern vaccines based on subunits of pathogens, such as purified proteins, are often unable to evoke strong immune responses. One of the most important challenges in the field is the selection of suitable adjuvants and delivery systems. Suitable adjuvants must enhance antigen specific immune responses, improve protection through stimulation of optimal types of immunity, and have low levels of adverse effects. Alum is currently the only adjuvant approved for human use by the United States Food and Drug Administration. Although alum has an excellent safety record, comparative studies showed that it is a poor adjuvant for recombinant proteins and DNA [18]. Additionally, alum is not effective for eliciting IgA antibody responses and some studies have even indicated that alum is associated with allergic reactions in some subjects [19].

Biodegradable and biocompatible polyesters, such as poly(lactic-co-glycolic acid) (PLGA), are one of the primary candidates for the development of microspheres as vaccines, because they have been used in humans for many years as suture material and as controlled-release delivery systems for peptide drugs [20]. One of the most attractive features of microspheres for vaccine development is their ability to control the rate of release of entrapped antigens over a longer period [21]. Ultimately, this may allow the development of single-dose vaccines through the preparation of microspheres that release entrapped antigens at times when booster doses of vaccines would normally be administered. The development of a single-dose vaccine would represent a significant advance towards the preparation of an ideal vaccine that would likely result in improved vaccine compliance, particularly for bovine mastitis.

The aims of this present study are 1) to express and purify a recombinant SEA as a vaccine candidate and to assess the efficiency of the PLGA adjuvant as a single-dose delivery system; 2) to establish a mouse model to investigate whether immunization with the PLGA-SEA vaccine could protect against *S. aureus *infection.

## Materials and methods

### Mice

Six to eight-week-old female specific-pathogen-free (SPF) mice were purchased from Merial-Vital Laboratory Animal Technology (Beijing, China). All animals were fed in germ-free isolators and experiments were in compliance with the guidelines of Beijing Municipality on the Review of Welfare and Ethics of Laboratory Animals approved by the Beijing Municipality Administration Office of Laboratory Animals.

### Anesthesia

Sodium pentobarbital (50 mg/kg) was given i.p. prior to all immunizations, collection of blood and bacterial challenge. A physical euthanasia method, cervical dislocation, was performed for collection of tissues, as previously described [22].

### Bacterial strain and chemical agents

Enterotoxigenic *S. aureus *(ATCC13565) was purchased from the Chinese National Center for Medical Type Culture Collections. The bacteria were cultured in tryptic soy broth (TSB) at 37°C with agitation at 120 rpm. *Escherichia coli *strains were grown in Luria-Bertani (LB) broth or on LB agar plates in the presence or absence of ampicillin (100 μg/mL).

### Glutathione-S-transferase (GST)-fusion SEA protein expression

For the amplification of the staphylococcal *sea *gene, primers (*sea*-1-774-F: 5'-CGGGATCCATGAAAAAAACAGCATTTACA-3'; *sea*-1-774-R: 5'-CCGCTCGAGTTAACTTGTATATAAATATATAT-3'; restriction enzyme sites underlined) were designed according to the published complete *sea *sequences (GenBank accession: AJ33122). The bacterial DNA was extracted by *EasyPure *Genomic DNAExtraction Kit (Transgen, Beijing, China). The polymerase chain reaction (PCR) assay was performed using 1 μL of DNA in a total reaction volume of 20 μL that contained 10 μL of Taq plus master mix (Transgen), 10 pmol of each of the two primers, and RNase-free H_2_O. The thermal cycling parameters used were as follows: 5 min at 95°C, followed by 30 cycles of 60 s at 94°C, 60 s at 45°C, and 60 s at 72°C, followed by extension for 10 min at 72°C. The reaction product was visualized by agarose gel electrophoresis and further confirmed by nucleotide sequence analysis. The amplified *sea *gene was ligated to the *Bam*HI/*Xho*I site of the pGEX-4 T-1 expression vector (GE Healthcare, Shanghai, China). The recombinant expression vector, pGEX-*sea*, was used to transform *E. coli *BL21 (DE3), and subsequently named pGEX-*sea*-BL21DE3.

The resultant recombinant *E. coli *strain was used to express and purify a Glutathione S-transferase (GST) fusion protein (designated rSEA) by affinity chromatography with Glutathione Sepharose 4 Fast Flow resin (Henghuibio, Beijing, China) according to the manufacturer's instructions. Protein concentration was determined by bicinchoninic acid (BCA) protein assay kit (Solarbio, Beijing, China) and analyzed by sodium dodecyl sulfate polyacrylamide gel electrophoresis (SDS-PAGE) and subsequent western immunoblot of GST fusion protein visualized using an enhanced chemiluminescence (ECL) Western Blot Kit (Beyotime Institute of Biotechnology, Jiangsu, China), according to the manufacturer's instructions. Mouse anti-GST tag monoclonal antibody (M&C Gene Technology, Beijing, China) was used as primary antibodies and horseradish peroxidase (HRP)-conjugated rabbit anti-mouse IgG (Jackson ImmunoResearch Laboratories, West Grove, PA, USA) as secondary antibodies, as described previously [23].

### Evaluation of the toxicity of recombinant SEA protein

To evaluate the toxicity and safety of the rSEA protein, groups of BALB/c mice (5 mice per group) were intraperitoneally (i. p.) administered either 1 mg, 100 μg or 10 μg rSEA protein per mouse. Mice in the negative control group were administered sterile phosphate-buffered saline (PBS; pH 7.4). The positive control group was administered i.p. 10 μg highly purified SEA (Sigma-Aldrich, Shanghai, China). Mice were sacrificed 7 days after injection, and livers were removed and fixed with 10% neutral-buffered formalin for histopathological evaluation, as previously described [24].

### Preparation and characterization of protein-loaded microspheres

PLGA microspheres (MS) containing rSEA were prepared by a water-in-oil-in-water (w/o/w) solvent extraction procedure, as previously reported [25]. Briefly, 100 mL of fusion protein (protein concentration, 5 mg/mL) was added to 500 mg of PLGA (50:50; Daigan Biological Materials, Shandong, China) in 10 mL of methylene chloride. The mixture was emulsified by sonication at output 300 W for 60 s to form the water-oil (w/o) emulsion. This emulsion was then added to 40 mL of 5% aqueous polyvinyl alcohol (PVA, MW 31,000; Sigma-Aldrich) solution to form the w/o/w emulsion. The organic solvent was stirred at a moderate speed at ambient temperature for 10 h. The rSEA-loaded MS were centrifuged at 5000 ×*g *for 45 min and then washed three times with double-distilled water. The MS were then lyophilized and stored at -20°C. Core loading of rSEA in MS was determined by digesting the MS and assaying the protein concentration [26]. Briefly, 20 mg of MS was suspended in 2 mL of 2.5% SDS/0.2 M NaOH solution, in which the SDS ensures the complete solubilization of the protein during polymer hydrolysis, and the resulting solution was then neutralized by the stepwise addition of 1 M hydrochloric acid. The amount of surface-associated protein was assessed by suspending the MS in PBS for 15 min, centrifuging the samples to sediment the MS, and then analyzing the supernatant for protein using the Bicinchoninic Acid (BCA) protein assay kit (Solarbio, Beijing, China).

MS size and surface morphology were examined by scanning electron microscopy (SEM; Hitachi S-3400; Hitachi, Japan). The total protein release dynamics was estimated using the BCA method [13,27,28]. Briefly, 100 mg of the MS were placed in test tubes containing 1 mL PBS (0.01 M; pH 7.4) and were incubated at 37°C with rotation. At 10-day intervals, the samples were centrifuged at 10 000 ×*g *for 10 min; the supernatant was collected and assayed for protein release using the BCA assay. The release study was continued after replacement with the same volume of fresh buffer.

### Immunization and determination of antibody responses by enzyme-linked immunosorbent assay (ELISA)

Groups of six female BALB/c mice (6-8 weeks old) were immunized i.p. with 100 μg of PLGA-rSEA, 100 μg of rSEA or 100 μg of empty PLGA. Control mice received 200 μL sterile PBS. Mice were restricted and were bled from the caudal vein (about 50 μL once) on days 0, 7, 14, 21, 28, 35, and 42 post-immunization. Serum IgG against SEA were assayed using an indirect ELISA [28]. Briefly, 96-well ELISA plates (Corning, Lowell, MA, USA) were coated with 100 μL/well of a 0.5 μg/mL highly purified SEA (Sigma-Aldrich) in carbonate buffer (0.01 M; pH 9.4) and incubated overnight at 4°C. The plates were thoroughly washed three times with PBS containing 0.05% Tween-20 (PBS-T; pH 7.4) and treated with blocking buffer (5% skim milk in PBS-T) at 37°C for 2 h. After washing, 100 μL of mouse serum (diluted 1:200 in PBS-T) was added to each well and incubated at 37°C for 1 h. After incubation, the plates were washed three times and 100 μL of HRP-conjugated goat anti-mouse IgG (Jackson ImmunoResearch Laboratories) was added to each well. The plates were incubated for 1 h at 37°C and washed three times. To develop the plates, 100 μL/well tetramethylbenzidine substrate solution was added to each well and incubated at 37°C for 15 min and stopped with 2 M H_2_SO_4_. The plates were read at 450 nm on an ELISA reader (Thermo Fisher Scientific Inc., Shanghai, China) and the results were reported as the OD_450 _of test well/OD_450 _of the control well (mouse serum from the control group) (P/N) [28].

### Challenge studies

Six weeks after immunization as above, five mice from each group were inoculated i.p. with *S. aureus *(ATCC13565) at the lethal dosage of 2 × 10^9 ^colony forming units (CFU; 2 LD_50_). Infected mice were monitored daily for mortality for 10 days.

### Bacterial burden and histopathological analysis

Two groups of mice (12 mice per group), immunized with either rSEA or PBS, were injected i.p. with sub-lethal doses of *S. aureus *(10^8 ^CFU, 0.1 LD_50_) and sacrificed 4 times on days 1, 3, 5, and 7 post-infection. Livers and spleens were aseptically removed and homogenized individually in 1 mL of sterile PBS. The viable bacterial levels were enumerated by plating the serially diluted homogenates on TSA medium. For histopathological evaluation, three immunized mice were sacrificed to excise the liver, spleen, and kidney on day 9 after infection. Organs were fixed in 10% neutral-buffered formalin and processed using standard histological methods as above.

### Statistical analysis

Data for the determination of bacterial burden and antibody level were expressed as mean ± standard error of the mean, and all the test groups were compared to the control group using the Student's *t *test. A *P*-value of < 0.05 was considered significant. Kaplan-Meier Log Rank methods were used for analyses of survival data. All statistics were performed using the Origin software (version 8.0; OriginLab, Northampton, MA, USA).

## Results

### Gene cloning and rSEA preparation

**Figure 1:**
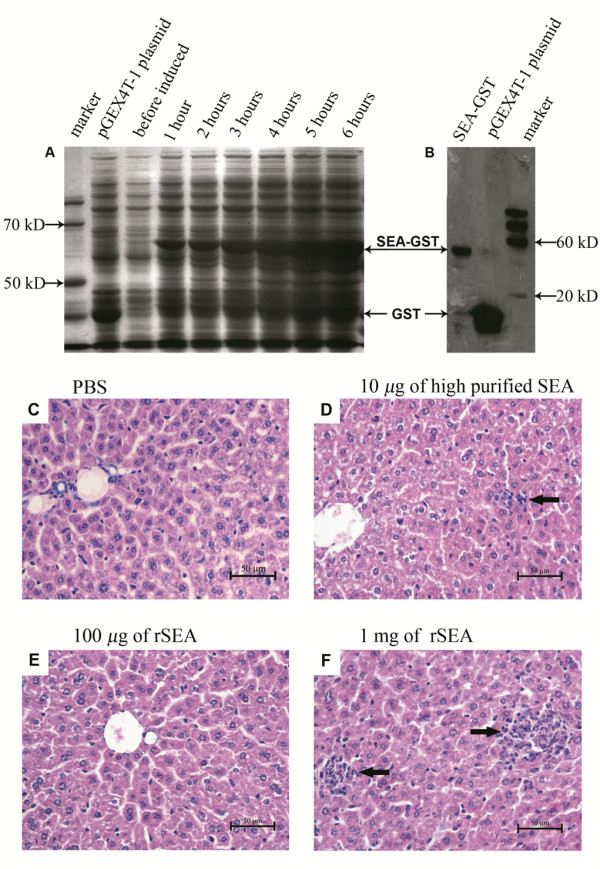
**SEA expression and toxicity analysis**. (A) SDS-PAGE for the rSEA induced by IPTG (1 mM) at different times; (B) Western immunoblot for rSEA protein, using anti-GST as the capture antibody; (C) histological imaging of liver sections from mice injected with PBS, (D) from mice injected with highly purified SEA protein, (E) low dose of rSEA experimental group, and (F) high dose of rSEA. Black arrows in D & F indicate the focal necrosis of hepatocytes and macrophage infiltration.

The *sea *gene isolated from *S. aureus *ATCC 13565 was sequenced to ensure that there were no mutations introduced during PCR amplification. SDS-PAGE showed that the GST-SEA fusion protein had a relative molecular mass of approximately 56 kDa expressed by the pGEX-*sea*-BL21DE3 strain (Figure 1a). The expressed protein was also demonstrated by western immunoblot (Figure 1b).

### Toxicity evaluation

In order to ensure that the fusion SEA was safe when administered as a vaccine, mice were injected i.p. with different dosages of the recombinant protein. None of the mice in the four groups showed any significant clinical symptoms during the experiment period. However, mice that received the high dose of rSEA (1 mg/mouse) (Figure 1f) and low dose of highly purified SEA group (10 μg/mouse) (Figure 1d) exhibited mild liver damage, by histological examination. Focal necrosis of hepatocytes and macrophage infiltration were obvious (Figure 1d, f). No pathological change was observed in other groups (Figure 1e). Therefore, the rSEA showed no significant toxicity in the low dosage range (< 100 μg) in mice.

### Characterization of PLGA microspheres

**Figure 2:**
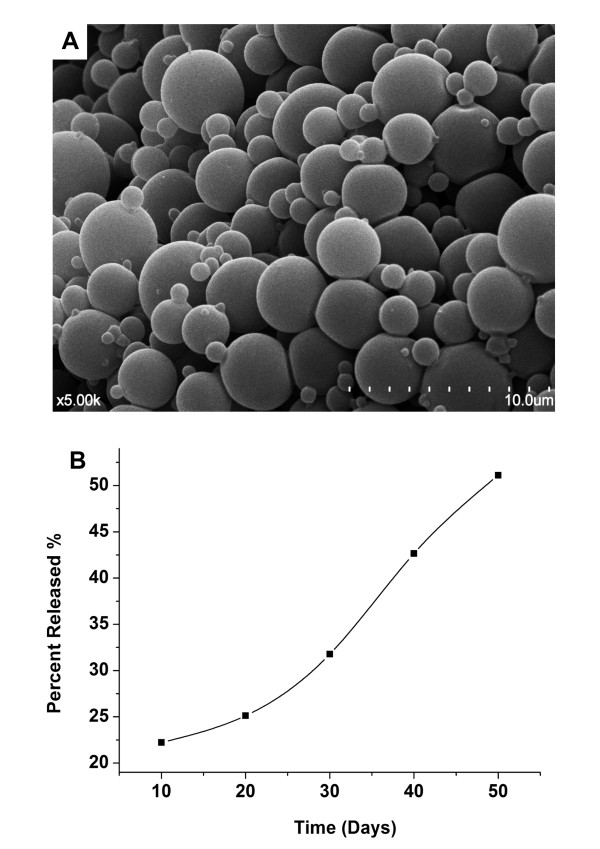
**Characteristics of the PLGA-rSEA**. (A) Scanning electron microscopy (SEM) photograph of PLGA-rSEA. Microsphere size, morphology, and surface appearance were examined by SEM. (B) Antigen-release kinetics. Percentage of released antigen was detected by BCA assay from day 10- 50.

The PLGA entrapped rSEA MS exhibited a size range from 1-5 μm in diameter with smooth surface under SEM (Figure 2a). The protein loading level of the MS was calculated by the amount of rSEA in a given weight of the polymer MS. The protein concentration in the MS was determined by BCA assay and showed a 78% loading level of the rSEA. The in vitro release kinetics of the rSEA was evaluated via BCA assay (Figure 2b), showing that rSEA was released over time. We found that rSEA had a sustained release from the MS after 20 days and released approximately 50% of loading dose in 50 days during polymer hydrolysis.

### Antibody response in mice

**Figure 3:**
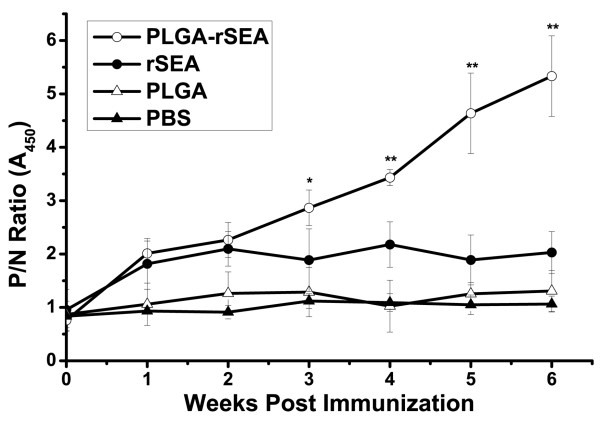
**Serum antibody analysis**. Antibody levels in mice serum. Sera were collected from vaccinated and unvaccinated controls as described in the text. The error bars indicate standard errors of the means for five mice per group. The asterisks indicate *P *values (*, *P *< 0.05; **, *P *< 0.01, compared with the PBS group).

The SEA-specific antibody responses induced by PLGA encapsulated rSEA were tested after i.p. administration. For comparison, groups of mice were immunized with non-adjuvanted rSEA and empty PLGA MS. Serum samples were collected at 7-day intervals. Antibodies developed against the SEA in serum were detected by indirect ELISA. From the fourth week after immunization, the MS vaccine induced significantly high antibody responses. Antibody response of the PLGA-rSEA group was higher than that of the rSEA group indicating that the MS adjuvant enhanced the immunogenicity of rSEA (Figure 3).

### Immuno-protection against bacterial infection

**Figure 4:**
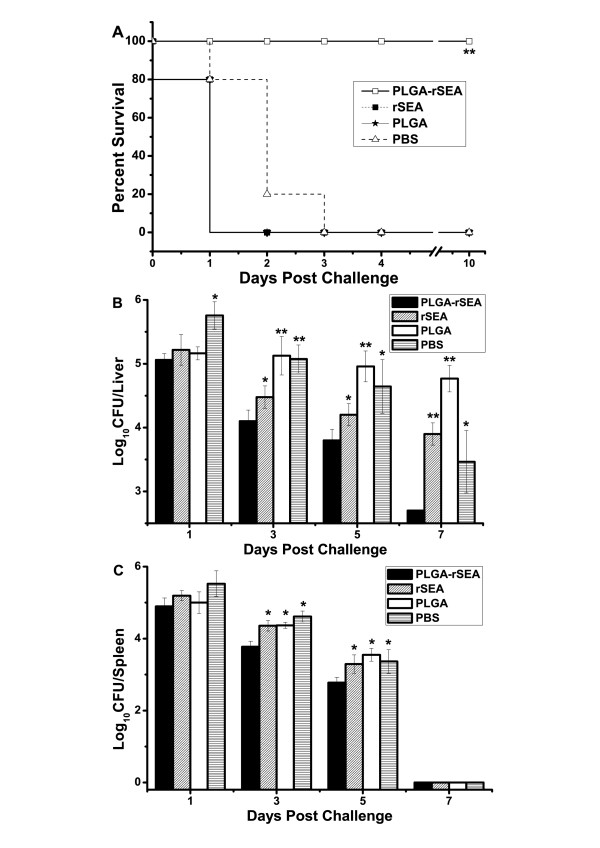
**In vivo evaluation of the PLGA-rSEA vaccine protection**. (A) Percent survival after infection; (B) In vivo growth of *S. aureus *in the liver after challenge; (C) In vivo growth of *S. aureus *in the spleen after challenge. BALB/c mice were vaccinated with PLGA-rSEA and then challenged i.p. with 10^8 ^CFU of *S. aureus *for bacterial clearance analysis (A and B) and 10^9 ^CFU lethal doses of *S. aureus *for percent survival analysis (C) (*, *P *< 0.05; **, *P *< 0.01, compared with the PBS group).

To determine whether the vaccine induced protection against *S. aureus *infection, groups of mice were inoculated with the MS entrapped with rSEA, or PBS, and then challenged with appropriate doses of *S. aureus*. When challenged with a lethal dose of *S. aureus*, 80% (4/5) of the unvaccinated mice died within 2 days post-infection (Figure 4a). However, 100% of the mice vaccinated with the microsphere vaccine survived.

It was next determined whether the increased survival in rSEA immunized mice was due to control of bacterial growth and dissemination during infection. Immunized- and sham-control mice were challenged with 10^8 ^CFU/mouse and bacterial burden in livers and spleens were determined. Significant differences in CFU were observed from day 3 pi (Figure 4b, c). No bacteria were recovered from spleens of all mice on day 7 (Figure 4c).

**Figure 5:**
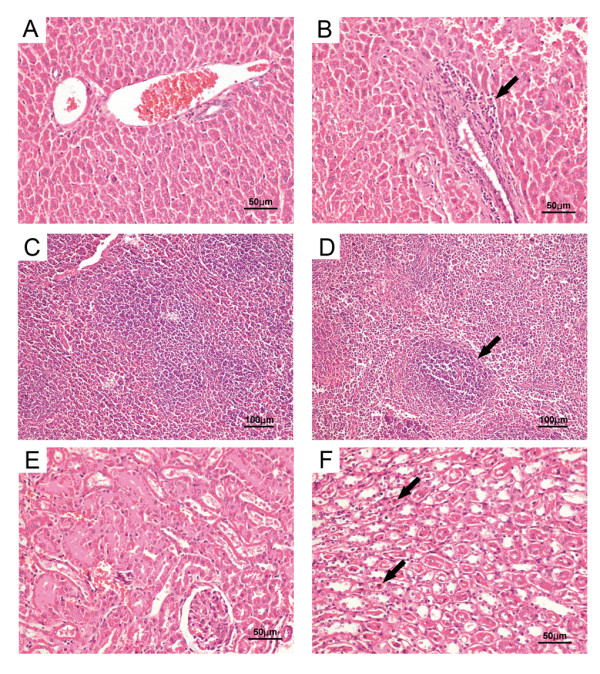
**Histological analysis of mice after challenge with *S. aureus***. (A) Liver histopathology from mice challenged with *S. aureus *after vaccination with PLGA-rSEA. (B) Liver histopathology of unvaccinated mice challenged with *S. aureus*. The black arrow indicates moderate infiltration of mononuclear cells around the central veins (C) Spleen histopathology of mice immunized with PLGA-rSEA. (D) Spleen histopathology of unvaccinated mice. (E) Kidney histopathology of mice immunized with PLGA-rSEA. The black arrow indicates erythrocytes and macrophages in the white pulp. (F) Kidney histopathology of unvaccinated mice. BALB/c mice were vaccinated and then challenged i.p. with 10^8 ^CFU of *S. aureus *six weeks after immunization. Mice were sacrificed on day 9 after challenge for this analysis. Tissues were stained with hematoxylin and eosin. Black arrows indicate the necrotic and scaled of the tubular structure.

Histopathological analyses were performed with vaccinated and unvaccinated mice 9 days after challenge (Figure 5). Vaccinated mice exhibited no obvious pathological change in the liver, spleen, and kidney (Figure 5a, c, e). However, the livers of sham-vaccinated mice showed moderate infiltration of mononuclear cells around the central veins (Figure 5b). In the spleen, lymphoid follicles were markedly decreased, and numerous erythrocytes and macrophages appeared in the white pulp (Figure 5d). Additionally, dilatation and hyperemia was demonstrated in Bowman's capsules of the kidney. Necrotic and scaled tubular structure was also observed (Figure 5f). These results show that using PLGA-SEA could protect mice against *S. aureus *infection.

## Discussion

*S. aureus *is a representative pathogen that can produce a number of potential virulence factors, including hemolysins, coagulase, leukocidin, enterotoxins, and TSST-1. The degree of severity of infection caused by *S. aureus*, which is related to these factors to some extent, may also vary among different strains of *S. aureus *[29]. It has been postulated that persistent infection with *S. aureus *is associated with an impaired immune response, which is also mediated by factors produced by *S. aureus*. Previous studies have investigated the role of *S. aureus *exosecretions in bovine udders by intracisternally inoculating them with sterile bacterial exosecretions from various strains of *S. aureus *[30,31].

SE have many distinct biological activities and can escape from normal host adaptive immune responses. For example, SE have the ability to bind to the major histocompatibility class II (MHC-II) molecules and specific V-segments of T-cell receptors (TCR) outside the binding groove that are associated with MHC-restricted immune system recognition of processed peptides. The binding of SAg to MHC-II and TCR stimulates abnormally large numbers of T cells. SE exert various deleterious effects, including induction of shock, cytokine induction, T-cell unresponsiveness and clonal deletion, differential stimulation of CD4^+ ^and CD8^+ ^T-cell subsets, and B-cell differentiation [30-32]. Although largely unconfirmed, in cases of bovine mastitis, there is a clear potential for SE to modulate immune responses and contribute to the virulence and persistence of *S. aureus *in cattle [17].

SE are believed to be related to common biological activities of toxins, which include pyrogenicity, immune response suppression, cytokine induction, proliferation of lymphocytes, and superantigenicity. Such biological activity plays an important role in lethal diseases, such as toxic shock syndrome. If the amino acid sequence of these functional structures is deleted or substituted with another amino acid sequence, SE can be used as vaccines or therapies in humans or animals. Bavari et al. [10] demonstrated that rSEA refers to SEA that has been mutated in the class II binding site and was safely used as a vaccine, which was also proved in a study performed by Collins et al. [33].

The N- and C-terminal peptides of SEA are important sites for interaction with MHC-II molecule or TCR. In the current study, the production of a recombinant expression vector containing the *sea *gene linked with the GST gene at the 5'-end suggests that the rSEA is altered leading to the loss of MHC-II and TCR binding sites. The reduction in toxicity compared to the native protein in this study suggests that it has reduced superantigenic activity, but further experiments are required to confirm that this is the case. Admittedly, further studies on rSEA toxicity through other aspects and in other animals, such as cattle and humans, should be performed for practical use of the rSEA based vaccines.

The use of polymeric MS as vaccine adjuvants and delivery systems has been investigated for their many advantages [34,35]. It has been demonstrated that a stronger immune response is elicited when an antigen is associated with MS compared with a soluble antigen alone. The delivery system controls the release of the entrapped antigens slowly and continuously. The most significant feature of a PLGA vaccine is the one-dose administration [36,37], and this was confirmed in the current study. The effect of particle size on immunogenicity is likely to be a consequence of enhanced uptake into the lymphatics and greater uptake into antigen-presenting cells for the smaller sized particles, since only MS < 5 μm were proven to be transported to the spleen [38]. The choice of the type of polymer is dependent on the actual needs of the antigen delivery system. The erosion of the polymer is dependent on two main parameters, molecular weight and monomer ratios; the higher the molecular weight of the polymer, the longer the time it will need to erode in vivo, and therefore will have a slower release. Also, the monomer ratios of lactic acid and glycolic acid in the polymer backbone affect erosion. Higher lactic acid content slows erosion rates in comparison to higher glycolic acid content. In the current study, polymers with molecular weight of 100 kDa, which is ideal for a two-month delivery time, were used to form the MS. This is consistent with other published work [20,27].

To prepare antigen entrapped MS, a certain amount of polymer is required to be present to ensure complete entrapment of the antigen within the particle. A 4-6% polymer solution is ideal for making small MS (< 10 μm) [25]; thus, we used a 5% polymer solution to prepare the MS. As observed by SEM (Figure 2a), the particle size of MS were between 1-5 μm.

Currently, vaccine efficacy against *S. aureus *is defined as an increase in the spontaneous cure rate [39-42]. In our study, antibody experiments showed a significantly high anti-SEA antibody titer giving protection against *S. aureus*. Lethal-dose experiments demonstrated that the PLGA-rSEA vaccine increased percent survival at a high dose (10^9 ^CFU/mouse) of *S. aureus *and histological tests proved that this vaccine caused less pathological damage at a lower dose (10^8 ^CFU/mouse) of this enterotoxigenic strain. Further, the PLGA adjuvant stimulated the host humoral immune response (Figure 5). However, no significantly increased differentiation of either CD4^+ ^or CD8^+ ^cells after vaccination were observed in our study according to flow cytometric assays (data not shown), although some published work proved that the cell-mediated immune response could be enhanced by PLGA adjuvant [43-46]. Therefore, further investigation is necessary to fully understand how the PLGA particles interact with the cell-mediated immune system. Due to the protective efficiency of the PLGA-rSEA MS vaccine developed in this study, we suggest that it should be possible to develop a subunit vaccination approach associated with PLGA MS for effective antibody-mediated protection against *S. aureus*. However, given the nature of *S. aureus *and the lessons learned from the recent failure of some emerging vaccines in phase III trials [47], it is clear that a multi-component vaccine is essential. Studies presented here indicate a positive approach to developing an efficient multi-component bovine mastitis vaccine that may contain different enterotoxins and other binding proteins associated with the high performance adjuvant, PLGA. This vaccine will be a promising strategy for preventing *S. aureus *caused bovine mastitis, because single dose administration presents significant superiority in treating dairy cows.

In conclusion, our strategy to combine rSEA with 5% PLGA polymer solution making 5 μm microspheres as a vaccine with a one-dose administration for two-month delivery proved to be a good inducer of immune responses against *S. aureus *infection in the mouse model. It produced high anti-SEA antibody titer and percent survival, as well as less pathological damage. These preliminary data are encouraging and underline the feasibility of a protective vaccine.

## Competing interests

The authors declare that they have no competing interests.

## Authors' contributions

BH conceived the study, and participated in its design, coordination, contributed to the analysis of the results and preparation of initial and revised manuscript versions. LBC, SL, ZFW participated in designing of the experimental strategy, carried out the glutathione-S-transferase (GST)-fusion SEA protein expression, evaluation of the toxicity of recombinant SEA protein, preparation and characterization of protein-loaded microspheres, immunization and determination of antibody responses, Bacterial burden and histopathological analysis; RLC carried out the challenge studies; JLS - participated in the design of the study and contributed to the preparation of the manuscript. All authors read and approved the final manuscript.
